# Augmenting Safety Planning With Text Messaging Support for Adolescents at Elevated Suicide Risk: Development and Acceptability Study

**DOI:** 10.2196/17345

**Published:** 2020-05-25

**Authors:** Ewa K Czyz, Alejandra Arango, Nathaniel Healy, Cheryl A King, Maureen Walton

**Affiliations:** 1 Department of Psychiatry University of Michigan Ann Arbor, MI United States; 2 Department of Psychology University of Houston Houston, TX United States; 3 Injury Prevention Center University of Michigan Ann Arbor, MI United States

**Keywords:** adolescents, suicide, text messaging, safety planning

## Abstract

**Background:**

Suicide is the second leading cause of death among adolescents. A critical need exists for developing promising interventions for adolescents after psychiatric hospitalization who are at a high risk of experiencing repeated suicidal behaviors and related crises. The high-risk period following psychiatric hospitalization calls for cost-effective and scalable continuity of care approaches to support adolescents’ transition from inpatient care. Text messages have been used to improve a wide range of behavioral and health outcomes and may hold promise as an accessible continuity of care strategy for youth at risk of suicide.

**Objective:**

In this study of 40 adolescents at elevated suicide risk, we report on the iterative development and acceptability of a text-based intervention designed to encourage adaptive coping and safety plan adherence in the high-risk period following psychiatric hospitalization.

**Methods:**

Adolescents (aged 13-17 years) who were hospitalized because of last-month suicide attempts or last-week suicidal ideation took part in either study phase 1 (n=25; 19/25, 76% female), wherein message content was developed and revised on the basis of feedback obtained during hospitalization, or study phase 2 (n=15; 11/15, 73% female), wherein text messages informed by phase 1 were further tested and refined based on feedback obtained daily over the course of a month after discharge (n=256 observations) and during an end-of-study phone interview.

**Results:**

Quantitative and qualitative feedback across the 2 study phases pointed to the acceptability of text-based support. Messages were seen as having the potential to be helpful with the transition after hospitalization, with adolescents indicating that texts may serve as reminders to use coping strategies, contribute to improvement in mood, and provide them with a sense of encouragement and hope. At the same time, some adolescents expressed concerns that messages may be insufficient for all teens or circumstances. In phase 2, the passage of time did not influence adolescents’ perception of messages in the month after discharge (*P*=.74); however, there were notable daily level associations between the perception of messages and adolescents’ affect. Specifically, higher within-person (relative to adolescents’ own average) anger was negatively related to liking text messages (*P*=.005), whereas within-person positive affect was associated with the perception of messages as more helpful (*P*=.04).

**Conclusions:**

Text-based support appears to be an acceptable continuity of care strategy to support adolescents’ transition after hospitalization. The implications of study findings are discussed. Future work is needed to evaluate the impact of text-based interventions on suicide-related outcomes.

## Introduction

### Background

As the second leading cause of death among adolescents in the United States [1], youth suicide is a major public health concern. At the same time, there are currently few effective interventions for suicidal adolescents and even fewer that have shown replicated efficacy [2-4]. Adolescents hospitalized because of severe suicidal ideation or suicide attempts are especially vulnerable to experiencing repeated suicidal crises in the postdischarge period [5-7], and there is a critical need to develop promising interventions to support adolescents’ transition from inpatient to outpatient care. Within the context of improving postdischarge outcomes for adolescents at elevated suicide risk, we describe the development of a technology-augmented (text-based) intervention focusing on strengthening adolescents’ motivation and self-efficacy to engage in adaptive coping after discharge.

### Technology-Augmented Interventions

Technological advances, particularly with respect to mobile phones, have increasingly allowed for the development of inexpensive and relatively accessible interventions with the potential to influence health outcomes and supplement established treatment and extend existing care services [8]. Moreover, the nearly ubiquitous mobile phone ownership among adolescents in the United States [9] has paved the way for mobile interventions to have wide reach and scalability. Mobile-based interventions, including text messaging, have been used to support a range of physical, behavioral, and mental health outcomes [8,10], with recent meta-analytic work indicating that text-based interventions have the potential to impact health behaviors in the short term and produce more lasting behavior change [11]. Although the majority of text-based interventions have targeted adult populations, there is emerging support for their promise in influencing youth outcomes [12,13]. For example, text-based interventions with youth have been associated with decreases in anxiety and depression [14], reductions in binge drinking [15], and decreases in smoking behaviors [16]. Further, text-based interventions for youth have shown acceptability and feasibility in the delivery of sensitive content, such as sexual health information [17] and violence prevention [18].

Mobile-based approaches may be well-suited to aid suicide prevention efforts, especially as previous research has pointed to caring contacts as being effective in reducing suicide deaths among high-risk populations [19,20]. Text-based interventions for individuals at increased suicide risk have incorporated a range of components, including caring contacts, resource information, self-help strategies, self-monitoring prompts, and links to additional psychoeducational tools [21-23]. Notably, text-based interventions specific to suicide prevention have been shown to be feasible and acceptable following acute crisis periods [24]. More recently, Berrouiguet et al [25] presented a case series from an ongoing trial of a brief contact text-based intervention for adults with a suicide attempt history and found that the intervention may have the potential to help connect individuals to crisis support services. Moreover, results from a randomized clinical trial indicated that veterans who received caring contacts via text messages were less likely to experience suicidal ideation and had fewer suicide attempts throughout the 1-year follow-up period [22]. Despite these promising findings, to our knowledge, text-based interventions aimed at reducing suicide risk have not been developed for or evaluated in high-risk adolescent samples.

### Text-Based Intervention for Adolescents Discharged From Psychiatric Hospitalization

Text-based interventions delivered after psychiatric hospitalization may hold promise as an accessible continuity of care strategy in the high-risk postdischarge period. Indeed, text messages have previously shown promise in supporting treatment maintenance or continuity of care during the transition from more intensive services for other health outcomes [26-29]. Here, our goal was to develop text-based support to reach adolescents at risk for suicide in the postdischarge period to explicitly encourage the use of adaptive coping strategies and safety plan adherence. Considered a best-practice approach for intervening with an individual at risk for suicide, a safety plan incorporates personalized coping strategies and resources for contacting social and personal support to mitigate a suicidal crisis. The clinical basis for safety plans is consistent with previous research showing that suicidal youth may engage in suicidal behaviors as a way of coping with distress [30] and tend to use less adaptive coping [31,32]. Empirical evidence for this suicide-specific intervention suggests that safety planning, combined with follow-up phone calls, was associated with a lower risk of suicidal behavior and improved treatment attendance among veterans [33]. A related crisis response planning intervention was similarly associated with a reduction in suicide attempts in an active duty military sample [34].

Despite the clinical value of safety planning, sustaining its use after psychiatric hospitalization, or using adaptive coping generally, may be challenging. Previous research on suicidal adolescents has shown that nearly 60% of discharged adolescents do not look at their safety plans in the 6 weeks after discharge, and more than half do not try to use coping strategies to manage self-harm thoughts (according to a presentation by Klaus N, PhD, October 27, 2011). In a pilot study of a safety planning intervention enhanced with motivational interviewing (MI), adolescents who received an MI-enhanced safety plan were more likely to use their safety plan when experiencing thoughts of suicide in the month after discharge compared to those who received standard safety planning [35], although safety plan use declined after discharge for both groups. Moreover, youth at risk for suicide report low confidence in their ability to use coping strategies recommended as part of safety planning [36]. As such, augmenting safety planning delivered during hospitalization with accessible follow-up strategies may promote its use, and thus increase its impact in the high-risk postdischarge period. This notion is consistent with the idea that mobile approaches may increase the salience and generalize the impact of interventions [37,38].

### Study Purpose

Guided by the overarching goal of improving postdischarge outcomes for adolescents at risk for suicide, the purpose of this study was to describe 2 initial phases in the development of a text-based intervention designed to encourage adolescents’ use of adaptive coping after psychiatric hospitalization and to sustain adherence to in-person safety planning. Thus, the intended goal of the text-based intervention was to encourage the use of individualized coping strategies identified as part of safety planning delivered during hospitalization, accessing different types of support, and using additional coping tools and resources. Given the value of youth feedback in developing text-based interventions [39-41], we iteratively sought the perspective of adolescents in the development and refinement process to maximize the saliency of text messages. In addition to describing the process of development, we report on the feasibility and acceptability of the text-based intervention as a continuity of care strategy promoting coping and safety plan use following discharge. The findings of this study are intended to inform the optimization of technology-augmented interventions for adolescents at elevated suicide risk.

## Methods

### Recruitment

Participants were 40 adolescents (aged 13-17 years) who were psychiatrically hospitalized because of last-month suicide attempts and/or last-week suicidal ideation and who took part in either phase 1 (n=25) or phase 2 (n=15) of the study (description in procedures). Exclusion criteria included severe cognitive impairment or altered mental status (eg, active psychosis or mania), transfer to residential placement, or unavailability of a legal guardian (ward of the state). Adolescents were also excluded from participation if they did not own a mobile phone with text messaging capability. Admitted eligible adolescents were approached for parental consent and adolescent assent. The study was approved by the participating university’s Institutional Review Board.

#### Phase 1

In Phase 1, 79% (27/34) eligible participants provided parental consent and adolescent assent. Of those, 25 adolescents completed all study procedures before discharge. The final phase 1 sample had a mean age of 15.24 (SD 1.36) years, with 76% (19/25) of the sample being female. The racial distribution was as follows: white (21/25, 84%), African American/black (1/25, 4%), and multiracial (3/25, 12%).

#### Phase 2

In phase 2, 83% (20/24) eligible adolescents provided assent and parental consent. Of those, 4 adolescents were discharged before completing baseline procedures, and 1 was not reached at follow-up, resulting in a sample of 15 adolescents. The final phase 2 sample had a mean age of 15.07 (SD 1.16) years. The sample was 73% (11/15) female with the following racial distribution: white (9/15, 60%), African American/black (3/15, 20%), and multiracial (3/15, 20%).

### Procedures

#### Initial Development

The development of text messages was guided by content area expertise in youth suicide prevention, theory, specifically self-determination theory (SDT) [42] and self-efficacy theory [43], and MI principles [44]. To promote behavior change, SDT places emphasis on encouraging intrinsic, rather than extrinsic, motivation by supporting individuals’ needs of autonomy, competence, and relatedness. Self-efficacy theory emphasizes that belief in one’s capability to succeed in a situation will ultimately shape behavior. Consistent with SDT and self-efficacy theory, MI uses a communication style that explicitly seeks to elicit and strengthen intrinsic motivation for change and support self-efficacy to achieve the change [44]. As such, we incorporated the principles and strategies of MI to support adolescents’ motivation and self-efficacy in using coping strategies after discharge. For example, messages incorporated autonomy-supportive language (eg, *if you want and it’s up to you*), affirming statements, and open-ended questions (eg, *what else do you find helpful?*) to encourage the reflection and generation of own ideas.

The bank of text messages was initially developed by coauthors (EC, NH, and CK) and included the following broad content areas: (1) self-efficacy to cope and keep self from suicidal action, (2) motivation to maintain safety, (3) tailored messages with personal coping strategies generated by the adolescent during safety planning (Phase 2 Procedures section), (4) suggestions for additional coping tips and strategies, (5) crisis resources, (6) encouragement to use personal safety plans, (7) affirmations, and (8) strengthening social connectedness. The messages included a combination of text, images, and/or links to Web-based resources or short videos. Table 1 includes example messages for each content area. In addition, supplemental (*extras*) messages were included to instill hope and facilitate engagement; these messages included inspirational quotes and humor. As described below, the process of refining the bank of messages occurred in 2 phases.

**Table 1:** Text message content and examples.

Content	Example messages
Self-efficacy	“Something to consider trying when you’re overwhelmed or down. Stop...Breathe...And think about how you got through difficult times before. You got this!”
Motivation	“You shared [individualized list of reasons for living] are some of your reasons for living. Reminding yourself of those can help when you need a boost. ”
Personal (tailored) coping strategies	“You shared [individualized list of 3 strategies from adolescent's safety plan] help you take a break from a problem or strong feelings. What else do you find helpful? You know yourself!”
Coping tips	“Paced breathing (deep breathing) can help with anxiety and stress. Practicing it when feeling calm can make it easier to use paced breathing in times of distress. If you want, see this video showing how to do paced breathing: [link to video]”
Crisis resources reminders	“It can be hard to do it alone. Consider reaching out to friends or family for support. Or think about calling confidential 24/7 hotline 800-273-8255.”
Affirmations	“We all have times our problems seem unbearable. And times we feel really alone. Even then, remember you got through it before. And that you matter!”
Support seeking; connectedness	“No man is an island.” Take a moment to reflect on people & places that comfort or lift you up. Try spending time with them or connect in your own way.”
Safety plan reminders	“When things get tough or painful, remember you have what it takes to cope! Remind yourself of your safety plan. It represents how strong you are. If you want, text PLAN to get an email with a copy of your safety plan you came up with when we first met.”
Extras^a^ (instilling hope)	Inspirational quotes were included with images representing the sentiment of the quote.

^a^Extras originally included humorous messages; however, these messages were considerably reduced following feedback (Results section).

#### Phase 1 Procedures

A total of 25 adolescents participated in phase 1 between April and June 2018. Participants completed the study procedures during hospitalization, which included completing a questionnaire using a tablet computer, wherein adolescents provided feedback on approximately 58 messages and responded to open-ended questions. The purpose of this phase was to revise the content and language of specific messages iteratively. Participants were compensated US $30 for their time and effort.

#### Phase 2 Procedures

In phase 2, 15 adolescents took part in the study between September 2018 and March 2019. The purpose of this phase was to test the bank of text messages after discharge and obtain participants’ feedback about their experience receiving the messages. Adolescents filled out an initial survey during hospitalization and subsequently participated in a safety planning session with a study counselor. The purpose of the safety planning meeting was to obtain information about adolescents’ individual coping strategies to personalize some of the text messages delivered after discharge. Starting on the first day after discharge, participants received 2 support text messages each day for 4 weeks. Text messages were sent using a digital platform, TextIt, which enables delivery of automated text messages in a predetermined sequence drawn from the message library, which also included personalized content. The information stored in the TextIt system included the phone number provided by the participant, contact preferences (eg, times of text messages), and specific message content scheduled to be delivered each day. The first message of the day was sent automatically (*push* message) in the morning at a preferred time previously identified by the participant. For the second message of the day, participants were prompted each afternoon, also at a time chosen by the participant, providing an option to request a message using a prespecified keyword (*pull* message). Thus, the second message of the day was sent only if requested by the participant.

Phase 2 participants were additionally asked to complete a brief survey each evening for 28 days after discharge, inquiring about their perception of the 2 support messages from the same day. A link to the survey, developed using a Qualtrics survey tool (SAP), was texted to participants’ phones each evening using the TextIt platform. Finally, phase 2 participants were asked to complete a 1-month follow-up survey over the phone, inquiring about adolescents’ overall perception about their experience receiving the support text messages. Participants were compensated US $10 for completing the baseline survey, US $4 for completing each of the daily surveys, and US $40 for the 1-month follow-up survey.

### Measures

#### Phase 1 Measures

##### Perception of Individual Messages

For each message, participants rated the extent to which they perceived a message positively (*How much did you like or dislike this message*?) using a 5-point scale ranging from *disliked* *a lot* to *liked a lot*. For each message, additional open-ended feedback was solicited by asking, *If you did not like something about the message or did not understand what we are trying to say, please give us feedback*.

##### Overall Feedback

After rating all messages, participants were asked to provide overall feedback using 5-point scales about their perception of the messages with regard to (1) adolescents’ interest in receiving text messages (from *not at all interested* to *very interested*), (2) extent to which receiving text messages like these might be helpful after leaving the hospital (from *very unhelpful* to *very helpful*), and (3) the extent to which a text-messaging program like this could help teens keep themselves safe, particularly if they have thoughts of suicide (from *no, definitely not* to *yes, definitely*). Additional open-ended feedback was sought regarding adolescents’ perception of why these messages might be helpful after hospitalization. Finally, adolescents were asked about their feedback regarding the type of message content they would prefer, the structure of the text-based intervention (eg, frequency of messages and duration of receiving the intervention), and possible concerns about privacy.

#### Phase 2 Measures

##### Perception of Individual Messages (Daily Survey)

Participants rated the extent to which they liked each message received daily using the same 5-point question as in Phase 1, ranging from *disliked a lot* to *liked a lot*. In addition, participants rated the extent to which each message was perceived as helpful using a 5-point scale from ranging from *very unhelpful* to *very helpful*.

##### Positive and Negative Affect (Daily Survey)

Positive and negative affect was rated on a 5-point scale, from *very slightly or not at all* to *extremely*. Items were adapted from the 10-item Positive and Negative Affect Schedule for Children (PANAS-C). The PANAS-C has good psychometric properties [45].

##### Hopelessness (Daily Survey)

Using a 4-point scale, from *strongly disagree* to *strongly agree*, hopelessness was measured with an item (*I see only bad things ahead of me, not good things*) derived from the 6-item brief Hopelessness Scale [46], which was adapted from the Hopelessness Scale for Children [47]. This item was selected because it had strong factor loading and an item-total scale correlation [47].

##### Overall Feedback (1-Month Follow-Up)

Using the same items as in phase 1, participants provided overall feedback pertaining to (1) adolescents’ interest in receiving text messages (from *not at all interested* to *very interested*), (2) the extent to which receiving text messages might be helpful to teens after leaving the hospital (from *very unhelpful* to *very helpful*), and (3) the extent to which the text-messaging program could help teens keep themselves safe (from *no, definitely not* to *yes, definitely*). Participants were provided with an opportunity to provide additional open-ended feedback.

In addition, participants were asked about their overall perception of text messages (*Overall, how much did you like or dislike the support text messages*?) using a 5-point scale ranging from *disliked a lot* to *liked a lot*. We also assessed the extent to which adolescents liked or disliked three broad types of messages: texts with coping tips or links to resources, texts with images that included affirmations or quotes, and texts with humorous content or memes.

Finally, adolescents were asked to provide open-ended feedback about their experience receiving messages with regard to logistics, including (1) what they thought about the timing (or time of day) they received messages, (2) what they thought about the number of support text messages, and (3) how long they thought the text-based intervention should last.

### Statistical Analysis

To obtain information about acceptability, frequencies, means, and SDs of feedback measures were summarized. Open-ended feedback was reviewed and coded. Specifically, a coding system was developed based on an initial review of the responses. The coding system summarized commonly mentioned responses for why text-based support may or may not be helpful. Participants’ responses were then coded by 2 independent coders, and any discrepancies were resolved by a third study team member. For daily data collected in phase 2, to explore the possible influence of daily affect and hopelessness on the perception of text messages in daily life, we utilized a series of linear mixed effects models. In each of these models, predictors were group mean-centered (score minus each participant’s own mean) to examine within-person effects, and each model also included the predictor’s corresponding group mean to simultaneously examine between-person effects. Descriptive statistics were obtained using SPSS (version 24) [48], whereas mixed effects models were conducted using SAS (version 9.4) [49].

## Results

### Phase 1 Results

#### Sample Characteristics

Over half of the adolescents (14/25, 56%) had at least 1 lifetime suicide attempt, with 40% (10/25) of the sample reporting a suicide attempt in the last month. All 25 adolescents experienced thoughts of suicide in the week before admission. Approximately one-third (9/25, 36%) of the phase 1 sample had at least 1 previous psychiatric hospitalization.

#### Phase 1 Feedback

Across the 58 messages that received individual ratings, participants perceived the majority of the messages positively (mean 4.26, SD 0.28). Consistently, as shown in Table 2, adolescents expressed that support text messages could be helpful after hospitalization (mean 4.67, SD 0.57), and most (17/24, 71%) indicated that receiving support text messages could probably or definitely help in preventing a postdischarge suicidal crisis.

**Table 2:** Overall feedback about text messages from phase 1 and phase 2.

Adolescent feedback			Phase 1 (n=24)		Phase 2 (n=13)
Extent to which text messages were perceived positively (1-5), mean (SD)			N/A^a^		4.31 (0.48)
Interest in receiving text messages (1-5), mean (SD)			4.33 (0.48)		4.08 (1.19)
Perception of text messages being helpful after hospitalization (1-5), mean (SD)			4.67 (0.57)		4.38 (0.65)
**Perception of text messages being helpful in preventing suicidal crises, n (%)**					
	No, definitely not	0 (0)		0 (0)	
	Probably not	0 (0)		1 (8)	
	Maybe	7 (29)		6 (46)	
	Probably	9 (38)		5 (38)	
	Yes, definitely	8 (33)		1 (8)	

^a^N/A: not applicable.

The three most commonly mentioned reasons for why text-based support may be helpful after discharge were the following: text messages serving as reminders to engage in coping behavior (n=9), for example,

teens would find this helpful because it would remind them of the tools they have and what they’ve learned;

messages providing encouragement (n=10), for example,

it helps remind yourself you can be safe even when it’s hard

it can help remind teens that they matter;

and messages contributing to an improvement in mood (n=10), for example,

something to lift their spirits even when they don’t really want to put effort into doing so

it may make someone’s day.

The most commonly cited reasons for why text messages could contribute to maintaining safety after discharge included providing ideas for coping (n=13), for example,

having resources to cope isn’t always easy to find and not everyone has the energy to look for them

there were numbers [to get] support and ideas to stay safe,

and encouragement and reminders of reasons for living (n=7), for example,

they would know they’re not the only ones going through this and there is hope and life is worth being here for

makes them think about the good things to look forward to...just the motivation to keep going, something to live for.

Some teens (n=5) cautioned that text messages may not be sufficient after discharge, for example,

it would be nice for [teens] to have support but if they are having suicidal thoughts they would probably need much more than [texts] to help them.

With regard to structuring the text-based intervention, most adolescents felt that messages should be sent at least once a day (n=12), with a notable number of youths expressing a preference for receiving messages twice a day (n=8). Approximately half (n=13) indicated that providing adolescents the ability to change the frequency of messages over time or offering the choice to request more messages should be incorporated. In terms of duration, most adolescents (n=10) felt that text messages should be provided for at least 1 month, with others suggesting 2 to 3 months (n=6) or even a year (n=2). As with frequency, many participants (n=11) felt that adolescents should have the option to personalize the duration of receiving the messages. None of the teens expressed privacy concerns about receiving support text messages postdischarge.

#### Changes and Considerations Following Phase 1

As part of reviewing each message, participants provided both ratings of individual messages and comments and suggestions for changes. We used this feedback to make changes to the initial bank of messages. We replaced lowest-rated messages with the content that adolescents indicated as being preferred. Specifically, the two most preferred content areas identified by adolescents were coping skills (n=14), with approximately half of the teens requesting links to additional information and encouraging and hopeful messages (n=9). Thus, we supplemented the message bank with these types of messages. In addition, as shown in Table 3, we used adolescents’ feedback to revise specific messages.

Consistent with the most common recommendation for the duration of text messages, we retained the initial idea to provide text messages in phase 2 for 1 month. We also structured the frequency of messages such that all adolescents received 1 message per day at the minimum. However, given that teens expressed the desire for autonomy and choice in receiving text messages, we provided an option in phase 2 to request an additional message each day and the ability to stop or restart receiving messages if desired.

**Table 3:** Examples of text message revisions.

Original message	Feedback	Revised message
(1) Think about people who supported you in the past. What did they say or do that you value? Consider saying *thanks* in your own way. Or see if you’d like to Pay It Forward: [website link added]	This could make someone upset if the people who supported them in the past are no longer with them. It could help, but because of my past and loss of everyone who cared, it makes me think bad thoughts.	“What *small* things have people said or done that brightened your day or helped you feel supported? See if you’d like to pay it forward: [website link added]”
(2) Everyone has triggers...situations, thoughts, emotions...that can signal a crisis. Knowing your triggers and how you’ll cope can help you stay in control.	Some people prefer not to use the word “trigger.” I'd try not to think of triggers.	“What helps you feel taken care of...physically, emotionally, or spiritually? You are worth taking the time and space to nurture yourself. Self-care can also help you stay in control of strong emotions or triggers. See if you like any of these self-care tips [website link added]”
(3) What are some things you care most about in life? Consider keeping reminders of what matters to you where you might see them (phone, wallet, room?).	The phrasing is a little off, it took me a minute to realize that you meant keep important things in your room wallet and phone and not the important things are the room, wallet and phone.	“When you need a boost, try re-focusing on what’s important to you...people, places, goals, things you value. Think about keeping reminders of what matters to you where you might see them. [image added expressing sentiment]”
(4) Sometimes we feel supported by being around certain people. Take a moment to think about people or places that comfort or lift you up.	I think you should encourage them to hang out with the person or people that are helpful.	“*No man is an island.* Take a moment to reflect on people & places that comfort or lift you up. Try spending time with them or connect in your own way.”
(5) How are you feeling today? Text back: GOOD, SOSO, or LOW [if LOW] Stay strong! Consider trying one of your coping tools that helps you feel better or reach out to someone. You have what it takes to get through this!	If low, give more direct instructions to feel better. Maybe give an example of a coping skill.	“[if LOW] Stay strong! Consider trying one of your coping tools, reach out to someone or see [link to website] for tips. You have what it takes to get through this!”

### Phase 2 Results

#### Sample Characteristics

About 60% (9/15) of adolescents in Phase 2 experienced at least one lifetime suicide attempt; 53% (8/15) of adolescents attempted suicide in the month before admission. All 15 adolescents reported suicidal ideation within the week of admission.

#### Daily Receptivity to Messages

Over the course of the 1-month follow-up (28 days), 15 participants responded to 61% (256/420) of daily surveys, and on average, participants completed 17.07 (SD 5.78) surveys. As part of these daily surveys, between 6 and 12 adolescents provided feedback on support text messages received on that day. The results indicate that the passage of time was not associated with the extent to which adolescents rated the messages favorably (B=.002, SE 0.01; *P*=.74) or as helpful (B=−.005, SE 0.01; *P*=.35). However, as shown in Table 4, positive and negative affect were associated with adolescents’ receptivity to messages. In particular, when adolescents reported higher levels of anger, relative to their own typical levels, support texts were rated less favorably; at the same time, perception of helpfulness was not associated with anger. When adolescents reported feeling happier, relative to their usual levels, they perceived messages as being more helpful, as did adolescents who generally reported feeling happier over the course of the 4 weeks as compared with those with less positive affect.

**Table 4:** Relationship between support text ratings and daily affect.

Variables^a^	Extent liked					Extent helpful			
	Within-person		Between-person		Within-person			Between-person	
	B (SE)	*P* value	B (SE)	*P* value	B (SE)		*P* value	B (SE)	*P* value
Happy	.03 (0.05)	.51	.27 (0.16)	.10	.09 (0.04)		.04	.27 (0.14)	.05
Angry	−.13 (0.05)	.01	−.06 (0.12)	.61	−.04 (0.04)		.37	−.08 (0.10)	.42
Miserable	−.02 (0.04)	.70	−.16 (0.13)	.20	−.07 (0.04)		.11	−.10 (0.11)	.35
Scared or anxious	.03 (0.04)	.48	.001 (0.10)	.99	−.04 (0.04)		.31	−.05 (0.09)	.56
Sad	−.03 (0.04)	.45	−.11 (0.13)	.37	−.06 (0.04)		.12	−.12 (0.11)	.26
Hopelessness	−.09 (0.08)	.24	−.23 (0.14)	.11	−.13 (0.07)		.08	−.20 (0.13)	.11

^a^All mixed effects models include random intercept; n=252 observations provided by 15 participants.

#### Phase 2 Feedback

Overall, 87% (13/15) adolescents completed the end-of-study assessment. As shown in Table 2, the perception of text messages was generally positive (mean 4.31, SD 0.48), and the messages tended to be perceived as helpful (mean 4.38, SD 0.65). Using open-ended responses, about half (n=7) of participants expressed that texts could be helpful by providing coping reminders and supporting adolescents’ transition from hospitalization (n=6), as illustrated by the following:

[you’re] transitioning back into the real world. And it’s good to have a reminder of the skills you learned while you were there. And it’s...like a cushion to help you with your transition back home.

...when you leave the hospital, a lot of the stuff that you learn there kind of goes out the window. And so, you know, to be reminded about your safety plan and things you might have learned there and things that make you happy is a really good way to kick-start recovery.

Some adolescents (n=6) also indicated that support text messages may be helpful in the postdischarge period by contributing to the improvement in mood and providing a sense of hope:

Because after you leave the hospital, you will feel most likely bad. So it’s nice to have that little cheer-up every day.

Getting those texts kind of made me feel better...on a day that was going rough, and I would look at it and do what it said...it just kind of made me feel better. Knowing that I can do this, and that people are there wanting to help me and get me better.

At the same time, 2 participants indicated that support delivered via an automated text messaging system may be limited:

[some people] could definitely benefit a lot from it, just some people don’t like automated text messages. So, like, texts from real people.

In terms of messages being helpful in preventing a suicidal crisis (see Table 2), nearly half (n=6) of adolescents indicated text messages could definitely or probably aid in the reduction of crises, for example,

definitely...just getting those tips and coping strategies, like little nudges, to remind myself of things I need to do to stay healthy and happy.

At the same time, some (n=5) cautioned that the influence of messages may vary based on individual circumstances, for example,

Even though it’s helpful, I’m not sure if for some people it would be enough.

Others (n=5) cautioned that the influence of messages may be limited because of the interaction being automated, for example,

it’s not someone actually talking to you twice a day.

#### Feedback About Logistics

The majority (n=10) of adolescents felt that at least 2 messages per day would be preferred. Specifically, whereas most (n=7) indicated that 2 messages per day were desired, for example,

two per day was the sweet spot.

Three teens suggested that additional messages per day may be helpful, for example,

I think it was a good amount [two per day], but there could be more. I mean, there’s room for more.

In terms of timing, the majority (n=7) felt that the timing of messages used in phase 2 (1 in the morning and 1 in the afternoon) was acceptable. Nevertheless, 3 adolescents suggested that changing the morning message timing on the weekends might make it more likely that teens will see those messages.

I was never up that early [on weekends]...I would have other notifications on my phone and I just wouldn’t notice it because I just wasn’t up.

Finally, with regard to the duration of text-based support following discharge, the majority (n=11) of adolescents indicated that messages should be sent for at least 1 month. Of those, 2 suggested that the duration could be extended to 2 or 3 months; 2 teens suggested that messages could be available indefinitely as long as there is an option to request stopping them.

#### Changes and Considerations Following Phase 2

We eliminated the majority of messages that included humor or memes. Adolescents tended to rate messages with humor as being more neutral (mean 3.69, SD 1.11), which was consistent with mixed open-ended feedback. In this context, 4 participants perceived these messages positively, for example,

It was nice to just get a laugh...Cause sometimes if I was having a bad time and something like that would pop up, you know it was kind of funny and I was just happy for a second. And just, like, realize that it’s not all bad.

On the other hand, 4 participants perceived these messages negatively, for example,

The one that looks like a meme...I found it more annoying than anything,

and 4 were neutral.

On the basis of participants’ overall acceptability of messages that included coping tips and resources (mean 4.08, SD 0.95) and messages with images that included affirmations or quotes (mean 4.38, SD 0.77), these messages were retained. Given the positive perception of messages with images and the possibility that visual representation of content could facilitate engagement, for example, one participant expressed the following:

[the images] were useful because they had coping mechanisms but they also weren't boring to look at,

We supplemented some of the messages, including coping tips, with an image representing the sentiment of the message.

Finally, we considered whether or not to retain the function of requesting the second message of the day (ie, *pull* messages). On average, adolescents requested a *pull* message on half the days (mean 14.73, SD 7.93), and as might be expected, the likelihood of requesting messages decreased over time (odds ratio 0.88, 95% CI 0.85-0.91; *P*<.001). This may have been because of habituation or boredom, which was noted by 2 participants, for example,

When I first started...I did it every day. But toward the end, it started to fall off. I just hadn’t looked at my phone...And then I didn’t bother because I wasn’t sure if it was going to let me see the message anyway.

In addition, at least 4 out of 13 adolescents interviewed at the end of the study indicated that they had inconsistent or limited phone access during the study. In the end, we elected to retain the *pull* function because it is consistent with providing adolescents with autonomy and because of the fact that, whereas 3 adolescents noted they would have preferred to receive the second message without requesting it, the majority (n=8) expressed that they liked this function, for example,

I liked the interaction,

and

I liked that it was kind of a surprise message and that it was optional.

## Discussion

### Principal Findings

The high-risk period following psychiatric hospitalization calls for continuity of care strategies that can support suicidal adolescents’ transition after acute services. Moreover, identifying cost-effective and scalable approaches with the potential to enhance existing suicide-specific interventions is imperative. Text messages have been used to improve a wide range of behavioral and health outcomes [8,10] and have been integrated into interventions targeting suicide prevention among adults [21-23]. This study examined the feasibility and acceptability of a text-based follow-up intervention for adolescents discharged from psychiatric hospitalization with a focus on encouraging coping behavior and safety plan adherence. To our knowledge, this is the first study to explore adolescents’ perception of text messages as a follow-up strategy to support youth at elevated suicide risk.

Consistent with previous studies [39-41], our findings support the importance of obtaining adolescents’ input in the development of text message content. Using a two-phase participatory process, we tested and refined the messages across eight primary domains. Overall, our results provide support for the acceptability of a text-based postdischarge intervention for teens at risk of suicide. A majority of eligible adolescents in phase 2 who were approached about receiving messages after discharge agreed to participate in the study, and during the course of the month-long intervention, none of these participants requested that messages be stopped being sent to them. Adolescents’ feedback indicated that, overall, messages were perceived positively, with the exception of messages incorporating humor. Adolescents expressed a preference for messages with coping tips and those with encouraging or hopeful content, particularly if they incorporated links and images. On the basis of this feedback, we adjusted the content of text messages and incorporated specific suggestions for changing the wording of specific messages. Moreover, messages were seen as having the potential to be helpful with the transition after hospitalization, with adolescents indicating that texts may serve as reminders to use coping strategies, contribute to improvement in mood, and provide teens with a sense of comfort and hope. At the same time, some adolescents expressed practical concerns that messages may not be sufficient or effective for some teens or circumstances. This underscores the fact that text-based interventions, even if perceived as acceptable and helpful, should be conceptualized as being adjunctive.

Another contribution of this study pertains to its exploration of factors impacting adolescents’ perception of messages in daily life. We observed that adolescents’ daily affect may influence their perceptions of messages on a given day. Adolescents rated messages less favorably, but not as less helpful on days they reported feeling more angry than usual. Increases in positive affect, on the other hand, were associated with messages being seen as more helpful. Over the month-long intervention, the passage of time did not influence messages being liked less or liked more, or the extent to which messages were seen as helpful. However, the passage of time was associated with a decreasing likelihood of adolescents requesting *pull* messages. Consistent with previous literature (see review [50]), engagement tends to decline over time and is a common challenge with mobile-based interventions. In this study, intervention engagement or *pulling* messages did not appear to be because of adolescents perceiving messages as being less helpful or less likable, as indicated by no time trend for these ratings. It is possible that, given the routine nature of message delivery (eg, sent at the same time of day), adolescents may have become desensitized to text notifications, subsequently impacting their motivation to request additional messages. Sending intermittent and more random prompts reminding adolescents that they can request additional messages and including a *teaser* in the prompt about the sort of message that could be requested on a given day, may increase engagement and add to the surprise element that some adolescents noted being important.

Related to the issue of timing, it will also be critical for future work to identify the optimal window of time to provide intervention prompts, which may be achieved with the application of real-time data (derived from ecological momentary assessments and passive sensing) that can help identify states of receptivity to mobile interventions to provide more personalized support [51,52]. Future interventions could also consider adding more elements of choice with regard to individualizing both the message content adolescents may find the most useful and the number of messages. Indeed, many adolescents in the study suggested that the frequency and duration of messages could be personalized. Lastly, it is important to highlight that, unlike in adults, there may be additional reasons that may impact adolescents’ ability to engage with mobile interventions, such as inconsistent phone access because of disciplinary reasons, and in the case of high-risk youth, loss of access because of hospitalization.

### Implications for Future Research

Although this study highlights the benefit of formative work with the target population in developing a text-based intervention and pretesting this intervention in the real world, our study did not explore the impact of receiving text messages on suicide risk-related outcomes. Text messages have shown promise in reducing suicidal behavior among adults [22]. However, the efficacy of text-based interventions has not been tested in adolescents. Even though adolescents in this study believed that these messages could be helpful after discharge, randomized controlled trials are needed to determine the extent to which text messages have an impact on actual outcomes. Although most adolescents in this study felt that receiving at least 1 message per day for at least 1 month would be adequate, another consideration for future research is whether or not different intensity or *doses* of messages may influence outcomes.

Finally, it will be important for future research to delineate the comparative benefits of different types of follow-up modalities in the postdischarge period. Among adults, integrating follow-up phone calls with a safety planning intervention after emergency department discharge was related to both decreases in suicidal behavior and treatment engagement across a 6-month follow-up period [33]. Additionally, previous research with adolescents has shown that receiving multiple phone contacts in the 90 days following psychiatric hospitalization was associated with reductions in suicidal behaviors and perceptions of improved confidence in the safety plan [53]. In the same study, 72% of adolescents engaged with at least one phone contact, but only 40% engaged with 3 or more phone contacts, which highlights some challenges in sustaining the intended dose of the intervention (6 phone contacts). Although not without its own challenges, as described previously, there nevertheless may be unique benefits inherent to text messages as a continuity of care approach. For example, text messages can be accessed at a later time and may be revisited repeatedly in the future, which could amplify their impact. Text messages can also be viewed discretely (eg, while surrounded by others) and at the discretion of the reader (eg, when it may be needed the most). Engagement with text messages may also require less effort and motivation, perhaps impacting utility when other methods are not being accessed. Finally, tools that allow text messages to be delivered automatically afford greater feasibility and scalability of text-based contact, particularly in low-resource settings.

However, there may be circumstances under which phone contacts are preferred or indicated. As raised by the adolescents in this study, the automated aspect of text-based interventions may be insufficient in some situations and for some adolescents at risk for suicide. There may also be circumstances under which the combination of both approaches may be most efficacious for some youth. For example, adjunctive text-based approaches may prove ideal as a low-cost, low-burden intervention that may, for some youth (eg, severe clinical presentations, not engaging or appearing to not sufficiently respond to a text-based intervention), benefit from being augmented by follow-up calls. To this end, our group is conducting a pilot intervention study examining different follow-up intervention strategies for youth discharged from psychiatric hospitalization that integrate text-based support and telephone contact.

### Limitations and Conclusions

Findings should be considered in the context of study limitations. Notably, although consistent with the demographics of the region and the inpatient adolescent population, our sample was primarily white and female, which limits the generalizability of the study results. Incorporating the perspective of more diverse adolescent samples will be imperative in future work to maximize the acceptability of text-based interventions for youth at risk of suicide. The sample size was consistent with similar studies [40,41]; nevertheless, the sample size limited our ability to carry out additional analyses examining text message feedback by subgroups (eg, age and sex). It is also important to note that the sample comprised help-seeking youth who consented to take part in the study, which may speak to their readiness to receive and engage with the intervention. Moreover, this study considered the acceptability of messages received for 1 month after discharge; whether the relatively positive perception of the text-based intervention would be sustained for a longer period is not known. Additional studies are thus needed to examine different approaches to augmenting suicide-specific interventions using text-based methods and examine text-based interventions in larger and more diverse adolescent samples. Despite these limitations, our findings provide useful information regarding text messages being seen as an acceptable approach for delivering follow-up support among adolescents at elevated suicide risk. The results suggest that adolescents were open to receiving text messages after discharge and perceived the messages as helpful in supporting their transition from psychiatric hospitalization. Future work is needed to evaluate the impact of text-based interventions, including different *doses* of text messages on suicide-related outcomes in the postdischarge period.

## Abbreviations

MI: motivational interviewing
PANAS-C: Positive and Negative Affect Schedule for Children
SDT: self-determination theory
